# Nicotine Suppresses Human Memory Th Cell Subsets With Preferential Effects on Central Memory Th Cells in an α7 Nicotinic Acetylcholine Receptor‐Dependent Manner

**DOI:** 10.1002/eji.70177

**Published:** 2026-04-02

**Authors:** Fatemeh Gholizadeh, Mehri Hajiaghayi, Niloufar Rahbari, Jennifer S. Choi, Samantha Heidt, Alexia Como, Maryam Kazerouni, Melika Kargar, Aude Pinard‐LaRoche, Steve C. C. Shih, Peter J. Darlington

**Affiliations:** ^1^ Department of Biology School of Health Concordia University Montréal Canada; ^2^ Department of Chemical and Materials Engineering Concordia University Montréal Canada; ^3^ Department of Medicine McGill University Montréal Canada; ^4^ Department of Psychology Concordia University Montréal Canada; ^5^ Department of Health Kinesiology & Applied Physiology, School of Health, Concordia University Montréal Canada; ^6^ Department of Electrical and Computer Engineering Concordia University Montréal Canada

**Keywords:** CD40L, *CHRNA7*, cytokines, memory T helper cells, NF‐κB p65

## Abstract

Memory T helper (Th) cells sustain protective recall responses but can also drive chronic inflammation, necessitating precise regulation of their effector programs. Although Th cells produce acetylcholine (ACh) and express nicotinic acetylcholine receptors (nAChRs), the contribution of nAChRs to human memory Th function across central (Tcm) and effector (Tem) subsets is poorly defined. We examined the effect of nicotine and GTS‐21, a compound previously described as targeting α7nAChR, on total memory Th cells and purified Tcm and Tem from healthy participants. Nicotine or GTS‐21 diminished IFN‐γ, IL‐4, and IL‐17A secretion, downregulated *TBX21*, *GATA3*, and *RORC*, and reduced NF‐κB p65 phosphorylation in total memory Th cells. Disruption of *CHRNA7* abolished nicotine‐mediated suppression but did not eliminate the inhibitory effects of GTS‐21. Within CCR7‐defined subsets, nicotine and GTS‐21 lowered Th1/Th2/Th17 frequencies in Tcm, but not in Tem. In purified subsets, nicotine suppressed IFN‐γ, IL‐4, IL‐17A, IL‐21, *BCL6*, and CD40L selectively in Tcm, whereas GTS‐21 suppressed them in both Tcm and Tem. Collectively, nicotine engages an α7nAChR‐dependent checkpoint that preferentially regulates Tcm responses, while GTS‐21 exerts broader suppressive effects not fully explained by α7nAChR loss. This cholinergic checkpoint in Tcm may limit Tfh‐associated help and pathogenic recall responses in immune‐mediated disease.

## Graphical abstract

Human memory Th cells were separated into Tcm (CD45RO^+^ CD62L^+^) and Tem (CD45RO^+^ CD62L^−^). Nicotine preferentially suppressed Th1‐, Th2‐, Th17‐, and Tfh‐associated programs in Tcm via an α7nAChR‐dependent mechanism, with minimal effect in Tem.

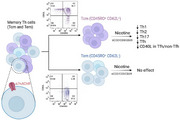

**Abbreviations**
ACh: acetylcholine
CD40L: CD40 ligand (CD154)
*CHRNA7*
: gene encoding α7 nicotinic acetylcholine receptor
nAChR: nicotinic acetylcholine receptor
Tcm: central memory T helper cells
Tem: effector memory T helper cells
Tfh: T follicular helper (cells)
Th: T helper (cells)

## 1 Introduction

The parasympathetic (cholinergic) branch of the autonomic nervous system plays a central role in regulating inflammatory responses through the release of its principal neurotransmitter, acetylcholine (ACh). Beyond neuronal input, immune cells themselves are capable of producing ACh, allowing for local cholinergic regulation of immune responses [1]. Notably, memory T helper (Th) cells are the major ACh‐producing population in the spleen [2]. ACh can regulate T cell function by modulating cytokine production, perturbing activation pathways, and altering chemokine receptor expression through muscarinic and nicotinic receptors [3, 4, 5]. Nicotinic acetylcholine receptors (nAChRs) are pentameric ligand‐gated ion channels composed of homomeric or heteromeric subunits [6]. Among these, the α7nAChR subtype exhibits both ionotropic and metabotropic signaling properties, functioning as an ion channel and signaling through G‐protein‐coupled pathways [7, 8]. ACh, acting through α7nAChR, can downregulate Th1 and Th17 differentiation and dampen cytokine release by activated Th cells [4, 9]. Regulation of Th cell responses is essential to defend against infection while avoiding pathological inflammation; when this balance is perturbed in clinical disease (e.g., rheumatoid arthritis and systemic lupus erythematosus), characteristic Th imbalances are observed [10, 11].

The cells orchestrate adaptive responses to non‐self‐antigens [12]. Th cell activation is driven in part by NF‐κB, specifically the canonical p65/p50 pathway, which drives cytokine transcription and supports immune homeostasis [13, 14, 15]. Naïve Th cells differentiate into specialized subsets such as Th1, Th2, Th17, T follicular helper (Tfh), and other subtypes of T cells [16]. Th1 cells, driven by *TBX21*, promote proinflammatory responses through IFN‐γ. Th2 cells, under the control of *GATA3*, produce IL‐4 to support humoral immunity. Th17 cells, defined by *RORC*, secrete IL‐17A to combat extracellular pathogens [17, 18, 19]. Tfh cells, marked by CXCR5 and *BCL6*, provide B cell help via CD40L and IL‐21 [20, 21]. Engagement of CD40 on B cells by CD40L on activated Th cells is essential for T‐dependent B‐cell activation and differentiation [22]. Non‐Tfh cells (CXCR5^−^) also express CD40L and can provide extrafollicular help [23], but Tfh signals support sustained germinal center responses [24]. After the pathogen is cleared, some effector Th cells transition into long‐lived memory Th cells, enabling rapid and robust secondary immune responses upon re‐exposure [25]. Memory Th cells are classically divided into central memory T cells (Tcm; CD45RO^+^ CCR7^+^ CD62L^+^), which retain lymphoid‐homing receptors with strong proliferative potential, and effector memory T cells (Tem; CD45RO^+^ CCR7^−^ CD62L^−^), which migrate to nonlymphoid tissues for immediate effector function [26, 27]. Memory Th cells help maintain immune homeostasis; however, when their activity increases and regulation diminishes, persistent cytokine production (e.g., IFN‐γ, TNF‐α, IL‐17) can drive chronic inflammation in diseases such as rheumatoid arthritis, multiple sclerosis, and psoriasis, making them targets for immunomodulation [28].

Nicotine is an agonist of nAChRs, including α7nAChR [29, 30], while GTS‐21 has been described as a partial agonist of α7nAChR in prior studies [31, 32]. In murine models, nicotine inhibited the differentiation of naïve Th cells into Th17 cells [3, 4], increased IL‐4, and dampened IFN‐γ production [4]. Nicotine reduced TNF‐α, IL‐1β, and IL‐12 and enhanced IL‐10 in mouse monocytes [33], reduced IFN‐γ, IL‐6, and TNF‐α in splenocytes [34], and suppressed IL‐17A in human peripheral blood mononuclear cells (PBMCs) [35]. Furthermore, GTS‐21 has been reported to inhibit TNF‐α, IL‐1β, and IL‐6 while enhancing TGF‐β production in murine microglia [36]. Notably, splenic memory Th cells released ACh during vagus nerve stimulation to suppress macrophage inflammation via α7nAChR signaling [2]. Although nicotinic signaling has been reported to dampen inflammatory responses in naïve/effector Th cells and innate immune cells, whether α7nAChR exerts similar regulatory control over human memory Th cells, which are long‐lived populations that sustain recall responses and contribute to chronic inflammation, remains unclear. Furthermore, given the functional divergence between Tcm and Tem memory subsets, it is unknown whether these populations respond equivalently to α7nAChR‐mediated signaling. In this study, we examined how α7nAChR‐associated nicotinic signaling shapes cytokine production, transcription factor expression, chemokine receptor profiles, and CD40L, comparing Tcm and Tem across Th1, Th2, Th17, and Tfh subsets.

## 2 Materials and Methods

### 2.1 Cell Preparation

The study was approved by the Concordia University Human Research Ethics Committee (file no. 30009292) and conducted in accordance with the Declaration of Helsinki guidelines. Participants were identified through self‐reporting during semistructured interviews. The study included healthy adult participants aged 18–76 years (median age: 23 years), with 56.6% female and 43.4% male, and representing diverse ethnic backgrounds. Exclusion criteria included age under 18 years, any self‐reported acute or chronic medical condition, current use of cholinergic agents or systemic corticosteroids, and current smoking or use of nicotine‐containing products. Participants were rescheduled if they reported vaccination or recreational drug use within the preceding 14 days. All participants provided written informed consent before blood collection. PBMCs were isolated as previously described [37], cryopreserved, and stored at −80°C short‐term until use.

**FIGURE 1:**
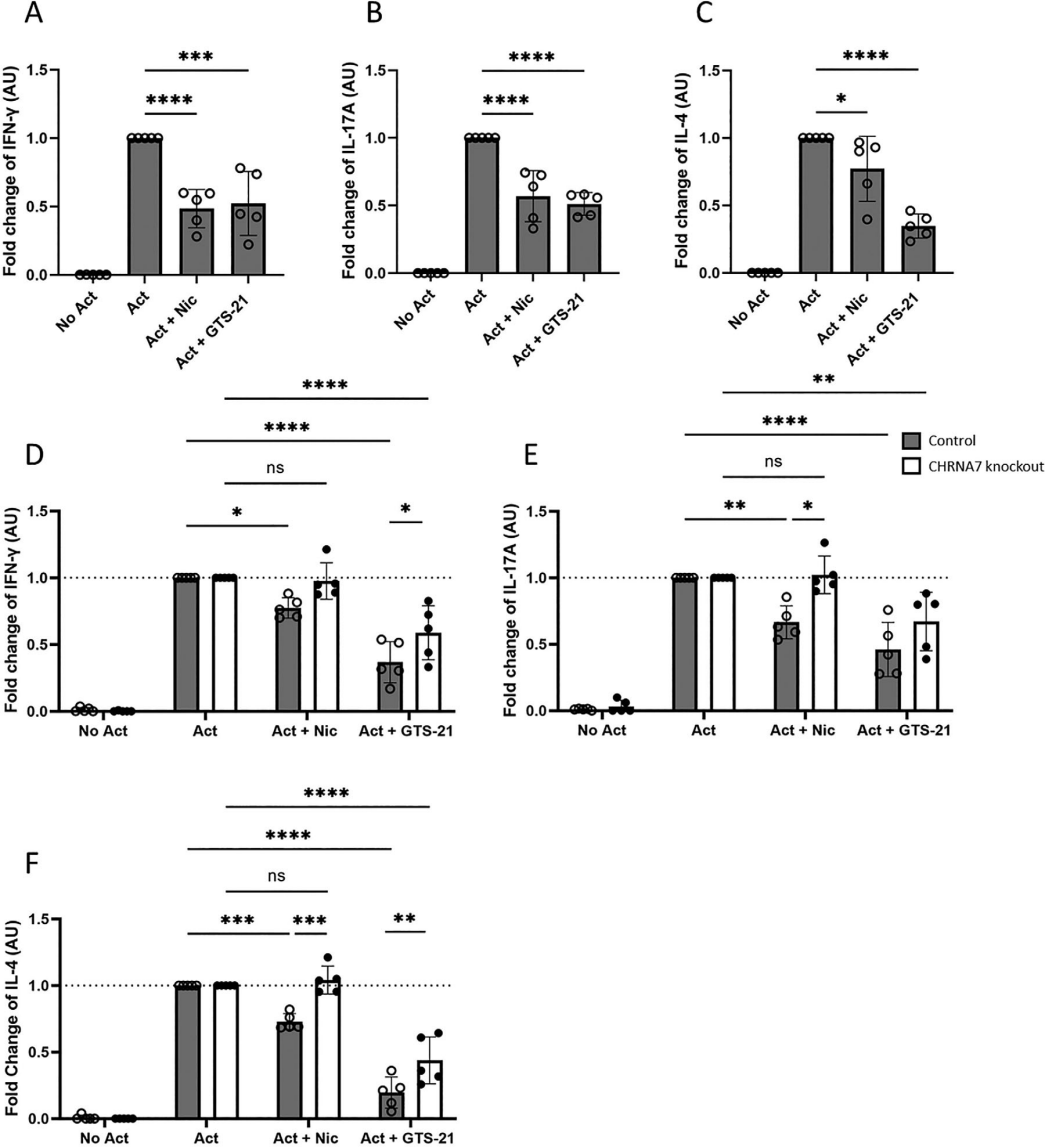
Effects of nicotine or GTS‐21 on cytokine secretion in total human memory Th cells and *CHRNA7*‐deficient memory Th cells. Memory Th cells were unstimulated (No Act), activated (Act), and activated in the presence of nicotine (Act + Nic) or GTS‐21 (Act + GTS‐21). Concentrations of (A) IFN‐γ, (B) IL‐17A, and (C) IL‐4 in culture supernatants were measured by ELISA. (D–F) Control (unedited, grey bars) and *CHRNA7*‐knockout (CRISPR‐edited, white bars) memory Th cells were cultured for 5 days under the same conditions, and secretion of (D) IFN‐γ, (E) IL‐17A, and (F) IL‐4 was compared between groups by ELISA. Data are presented as mean ± SD from five independent participants. Group comparisons were analyzed using one‐way or two‐way ANOVA with Tukey's post hoc test for multiple comparisons.

**FIGURE 2:**
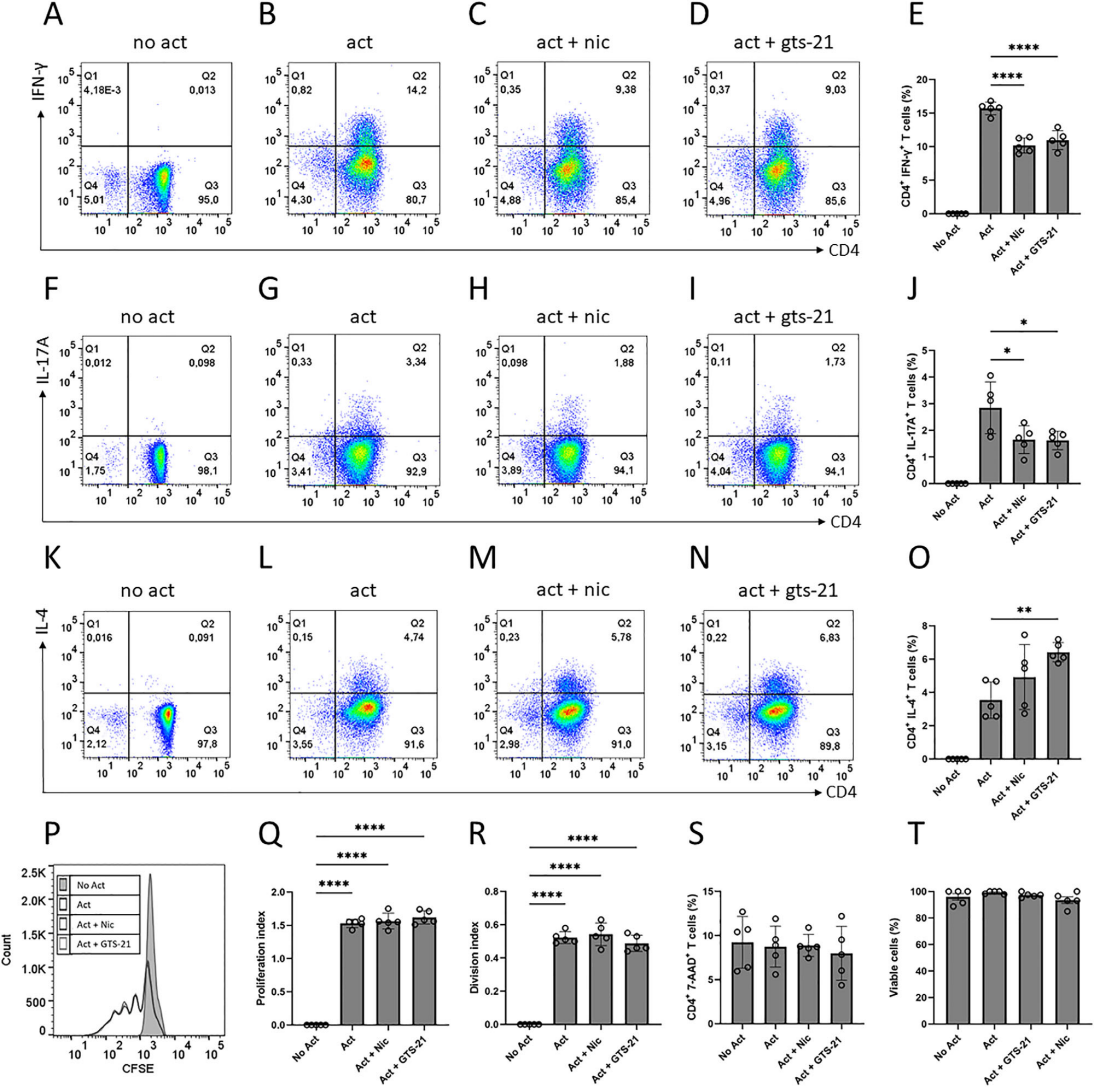
The effect of nicotine or GTS‐21 on cytokine expression, proliferation, and viability of total human memory Th cells. Total human memory Th cells were unstimulated (No Act), activated (Act), and activated in the presence of nicotine (Act + Nic) or GTS‐21 (Act + GTS‐21). The cells were examined for surface expression of CD4 and intracellular expression of IFN‐γ, IL‐17A, and IL‐4. Representative flow cytometry plots showing (A–D) CD4^+^ IFN‐γ^+^, (F–I) CD4^+^ IL‐17A^+^, and (K–N) CD4^+^ IL‐4^+^ cells, with (E, J, O) showing the corresponding quantification of cytokine‐producing memory Th cells. To evaluate the effect of nicotine or GTS‐21 on proliferation, total memory Th cells were labeled with CFSE. (P) Representative histograms of CFSE fluorescence intensity for each condition, with bar graphs displaying the (Q) proliferation index and (R) division index. (S) Cell viability was assessed using 7‐AAD staining with quantification of dead memory Th cells, and (T) total cell numbers were determined using trypan blue exclusion. Data are presented as mean ± SD from five independent participants. Group comparisons were analyzed using one‐way ANOVA with Tukey's post hoc test for multiple comparisons.

**FIGURE 3:**
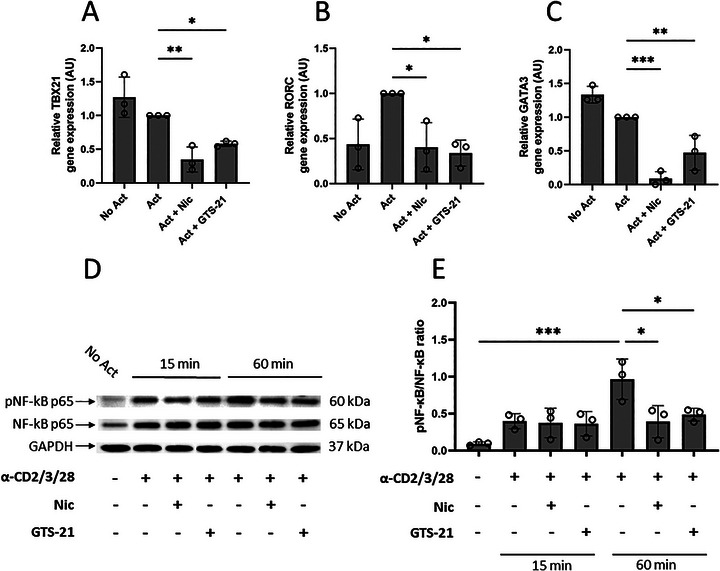
The effect of nicotine or GTS‐21 on the mRNA expression of *TBX21*, *RORC*, *GATA3*, and phosphorylation of NF‐kB p65 in total memory Th cells. Memory Th cells were unstimulated (No Act), activated (Act), and activated in the presence of nicotine (Act + Nic) or GTS‐21 (Act + GTS‐21). The relative mRNA expression levels of (A) *TBX21*, (B) *RORC*, and (C) *GATA3* were assessed by RT‐qPCR. (D, E) For NF‐κB analysis, cells were cultured for 15 or 60 min under the same conditions, and western blot was performed on lysates to detect phosphorylated Ser529 NF‐κB p65 (pNF‐κB p65) and total NF‐κB p65. (D) Representative western blot showing pNF‐κB p65 and total NF‐κB p65, with GAPDH as a loading control. (E) Quantification of pNF‐κB p65 levels normalized to total NF‐κB p65. Data are presented as mean ± SD from three independent participants. Group comparisons were analyzed using one‐way ANOVA with Tukey's post hoc test for multiple comparisons.

**FIGURE 4:**
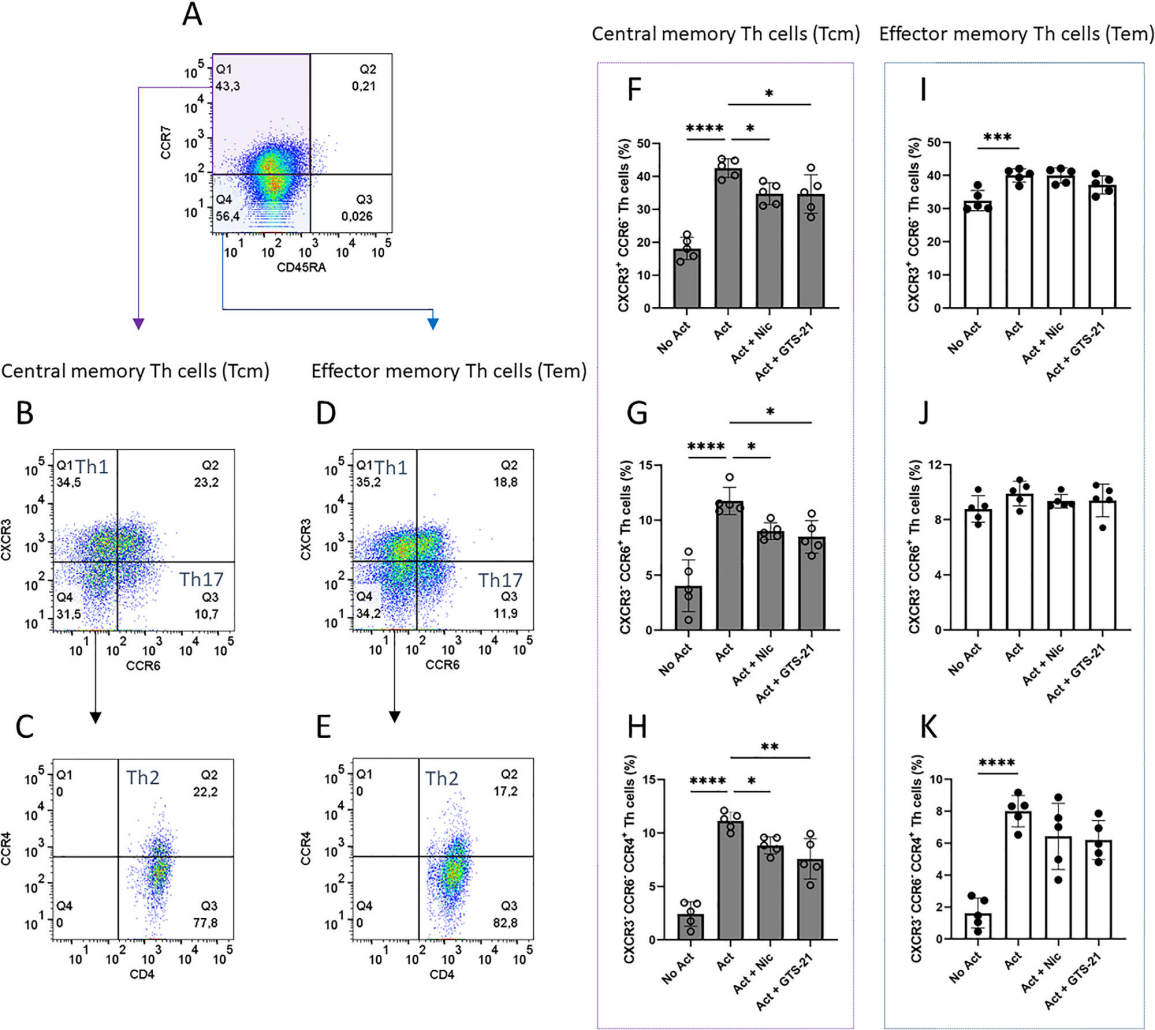
The effect of nicotine or GTS‐21 on chemokine receptor‐defined Th subsets within central and effector memory Th cells. Memory Th cells were unstimulated (No Act), activated (Act), and activated in the presence of nicotine (Act + Nic) or GTS‐21 (Act + GTS‐21). (A) Gating strategy identifying Tcm (Q1: CD4^+^ CD45RA^−^ CCR7^+^) and Tem (Q4: CD4^+^ CD45RA^−^ CCR7^−^). Within Tcm, (B) Th1 (Q1: CXCR3^+^ CCR6^−^), Th17 (Q3: CXCR3^−^ CCR6^+^), and (C) Th2 (Q2: CXCR3^−^ CCR6^−^ CCR4^+^). Within Tem, (D) Th1 (Q1: CXCR3^+^ CCR6^−^), Th17 (Q3: CXCR3^−^ CCR6^+^), and (E) Th2 (Q2: CXCR3^−^ CCR6^−^ CCR4^+^). (F–H) Quantification of Th1, Th17, and Th2 subsets within Tcm, and (I–K) Quantification of Th1, Th17, and Th2 subsets within Tem. Data are presented as mean ± SD from five independent participants. Group comparisons were analyzed using one‐way ANOVA with Tukey's post hoc test for multiple comparisons.

**FIGURE 5:**
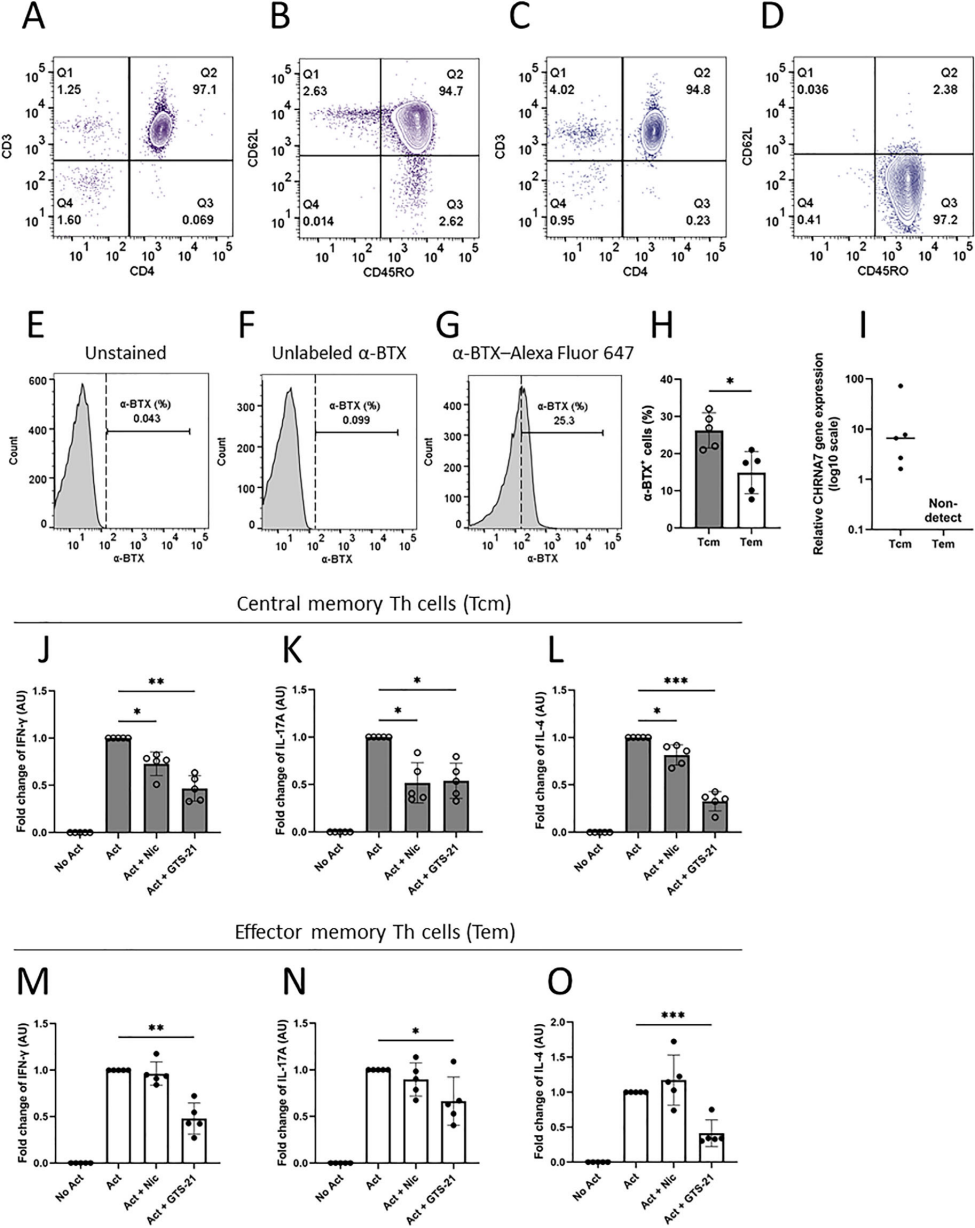
Baseline expression of α7nAChRs and the effect of nicotine or GTS‐21 on cytokine secretion in separated central and effector memory Th cells. Magnetic immuno‐panning was used to separate Tcm and Tem based on consistent CD45RO expression and differential CD62L expression. (A, B) Gating strategy confirming purity of Tcm (CD3^+^ CD4^+^ CD45RO^+^ CD62L^+^) and (C, D) Tem (CD3^+^ CD4^+^ CD45RO^+^ CD62L^−^). (E–G) Representative histograms illustrate the gating strategy used to define α‐BTX^+^ cells, based on unstained, unlabeled α‐BTX and α‐BTX‐Alexa Fluor 647‐labeled controls; histograms were generated from cells gated on the corresponding Tcm or Tem populations. (H) Quantification of the frequency of α‐BTX^+^ cells in Tcm and Tem. (I) Relative *CHRNA7* transcript abundance in freshly isolated Tcm and Tem, normalized to *PPIA* (log_10_ scale); samples with undetermined Ct values were considered nondetect. Tcm and Tem were unstimulated (No Act), activated (Act), and activated in the presence of nicotine (Act + Nic) or GTS‐21 (Act + GTS‐21). Concentrations of IFN‐γ, IL‐17A, and IL‐4 were measured by ELISA in (J–L) Tcm and (M–O) Tem from cell culture supernatants. Data are presented as mean ± SD from five independent participants. The frequency of α‐BTX^+^ cells between Tcm and Tem was analyzed using a paired two‐tailed Student's *t*‐test, while group comparisons for cytokine data were analyzed using one‐way ANOVA with Tukey's post hoc test.

**FIGURE 6:**
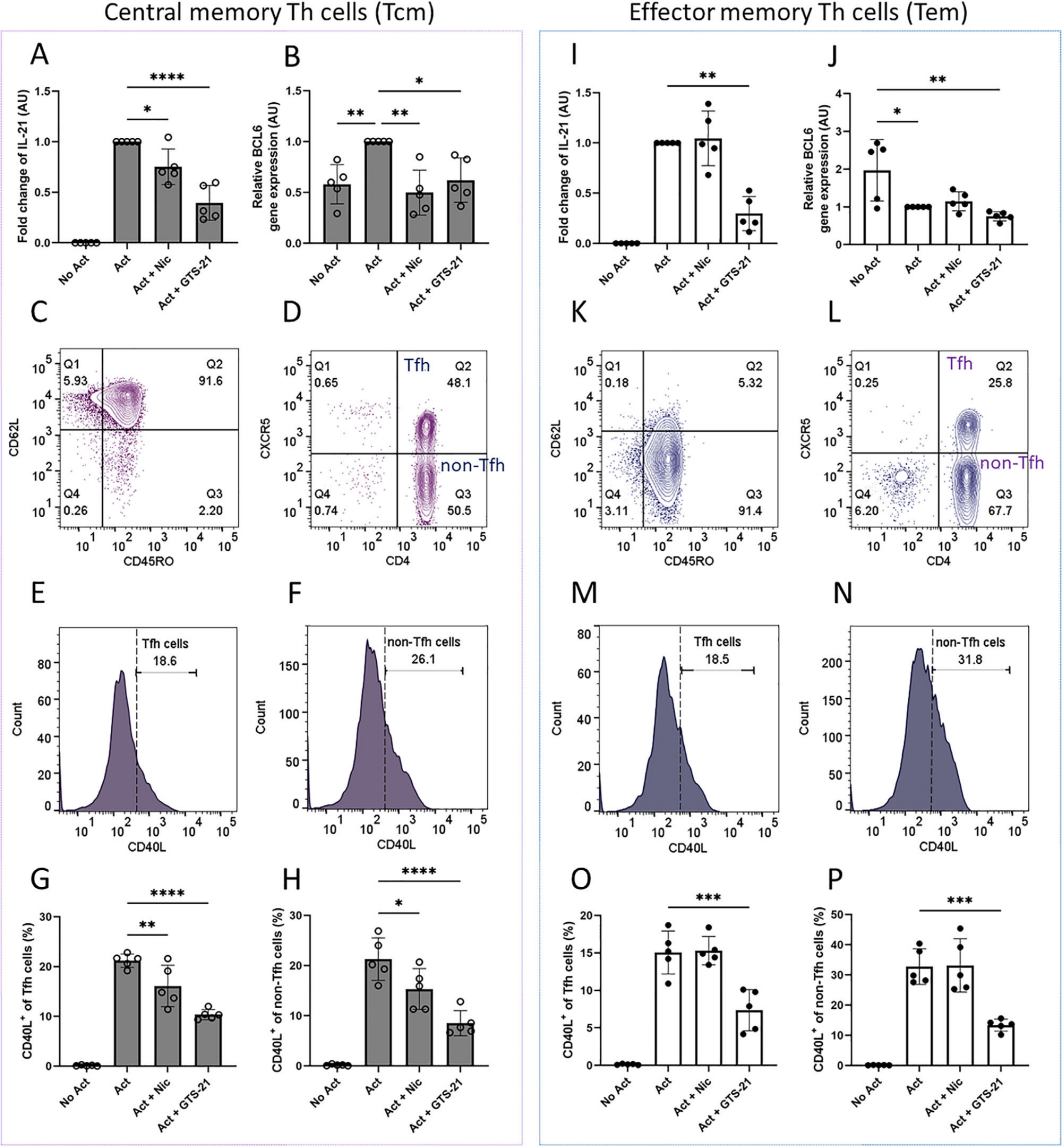
The effect of nicotine or GTS‐21 on IL‐21, *BCL6*, and CD40L in separated central and effector memory Th cells. Tcm and Tem were left unstimulated (No Act), activated (Act), or activated in the presence of nicotine (Act + Nic) or GTS‐21 (Act + GTS‐21). Tcm (A‐H; CD45RO^+^ CD62L^+^): (A) IL‐21 secretion; (B) *BCL6* mRNA; (C, D) gating of CD45RO^+^ CD62L^+^ cells and subdivision into CXCR5^+^ Tfh vs. CXCR5^−^ non‐Tfh; (E, F) representative CD40L histograms for Tfh and non‐Tfh; (G, H) frequencies of CD40L^+^ Tfh and CD40L^+^ non‐Tfh. Tem (I–P; CD45RO^+^ CD62L^−^): (I) IL‐21 secretion; (J) *BCL6* mRNA; (K, L) gating of CD45RO^+^ CD62L^−^ cells and subdivision into CXCR5^+^ Tfh vs. CXCR5^−^ non‐Tfh; (M, N) representative CD40L histograms; (O, P) frequencies of CD40L^+^ Tfh and CD40L^+^ non‐Tfh. Data are presented as mean ± SD from five independent participants. Group comparisons were analyzed using one‐way ANOVA with Tukey's post hoc test for multiple comparisons.

For most experiments shown in Figures 1, 2, 3, 4, total memory Th cells were isolated using the EasySep Human Memory CD4^+^ T Cell Enrichment Kit (STEMCELL Technologies, Vancouver, Canada) according to the manufacturer's instructions. Purity was assessed by flow cytometry after staining for CD3, CD4, CD45RA, and CD45RO, as previously described [37]. For experiments directly comparing central and effector memory populations (Figures 5 and 6), CD3^+^ CD4^+^ CD45RO^+^ CD62L^+^ central memory and CD3^+^ CD4^+^ CD45RO^+^ CD62L^−^ effector memory Th cells were further purified using the EasySep Human Central and Effector Memory CD4^+^ T Cell Isolation Kit (STEMCELL Technologies), following the manufacturer's protocol. Purity of each subset was confirmed by flow cytometry.

### 2.2 Memory Th Cell Culture and Stimulation

Memory Th cells were suspended in RPMI 1640 supplemented with 10% fetal bovine serum (FBS), 100 units/mL penicillin, 100 µg/mL streptomycin, and 2 mM GlutaPLUS (Wisent Bioproducts) at 37°C under a humidified atmosphere with 5% CO_2_. Cells were plated at 1.5 × 10^6^ cells/mL in 96‐well U‐bottom tissue culture plates (Fisher Scientific) and activated for 5 days with 3.125 µL/mL ImmunoCult Human CD3/CD28/CD2 T Cell Activator (StemCell Technologies) in the presence or absence of 10 µM nicotine (Cayman Chemical, distributed by Cedarlane, Burlington, Canada; see Figure S1) or 10 µM GTS‐21 (Cayman Chemical, distributed by Cedarlane). For short‐term assays, cells were stimulated for 6 h to assess CD40L expression, or for 15 and 60 min to measure NF‐κB p65 phosphorylation.

### 2.3 ELISA

Supernatants were assayed for IFN‐γ and IL‐4 (BD Biosciences, USA) and for IL‐17A and IL‐21 (Thermo Fisher Scientific, Burlington, Canada) according to manufacturers’ protocols. The substrate used to detect IL‐4 and IFN‐γ was the Ultrasensitive ELISA substrate for horseradish peroxidase, obtained from Applied Biological Materials Inc. Absorbance was measured at 450 nm with a microplate reader (Bio‐Tek, BD Biosciences, Mississauga, Canada), and the reading at 570 nm was subtracted.

### 2.4 Flow Cytometry

The purity of total memory Th cells, proliferation assays using CFDA‐SE labeling, and viability assessment with 7‐aminoactinomycin D (7‐AAD) were performed as described previously [5]. Regarding intracellular cytokine staining, memory Th cells were restimulated on day 5 with 50 ng/mL phorbol 12‐myristate 13‐acetate (PMA), 500 ng/mL ionomycin, and 10 µg/mL brefeldin A (Sigma‐Aldrich) for 5 h at 37 °C to induce cytokine production and inhibit secretion. Cells were then centrifuged (493×*g*, 7 min, room temperature), and the supernatant was discarded. Cells were surface‐stained with anti‐CD4‐APC (clone RPA‐T4, BD Biosciences) for 30 min on ice, then fixed and permeabilized using the BD Pharmingen Transcription Factor Buffer Set (BD Biosciences). Cells were subsequently stained for intracellular cytokines using anti‐IFN‐γ‐BV421 (clone B27, BD Biosciences), anti‐IL‐17A‐PE (clone BL168, eLabscience from Cedarlane), and anti‐IL‐4‐PE‐Cy7 (clone 8D4‐8, Thermo Fisher Scientific). Isotype‐matched controls were included to define background fluorescence and validate antibody specificity as previously described [5].

To analyze chemokine receptors and Tfh‐associated markers, cells were harvested, washed with PBS, incubated on ice for 30 min with fluorochrome‐conjugated antibodies in staining buffer (PBS with 2% FBS and 0.1% sodium azide), washed, and resuspended in fresh staining buffer for acquisition. Chemokine receptor staining included labeling with anti‐CD4‐APC, anti‐CD45RA‐FITC (clone HI100, Thermo Fisher Scientific), anti‐CCR7‐BV421 (clone 2‐L1‐A, BD Biosciences), anti‐CXCR3‐PerCP‐Cy5.5 (clone 1C6, BD Biosciences), anti‐CCR6‐PE (clone 11A9, BD Biosciences), and anti‐CCR4‐PE‐Cy7 (clone 1G1, BD Biosciences). For detection of Tfh‐associated markers, cells were stained with anti‐CD62L‐PE‐Cy7 (clone DREG56), anti‐CD45RO‐FITC (clone UCHL1, BD Biosciences), anti‐CXCR5‐APC (clone MU5UBEE), anti‐CD4‐PE (clone RPA‐T4, BD Biosciences), and anti‐CD40L‐PerCP‐eFluor 710 (clone24‐31). Antibodies were used at predetermined optimal concentrations according to the manufacturers’ protocols. Isotype controls were included to guide gating boundaries (Figures S3A–F and S4A,B).

The purity of Tcm and Tem subsets was verified by surface staining with anti‐CD3‐BV421 (clone OKT3, BD Biosciences), anti‐CD4‐APC, anti‐CD45RO‐FITC, and anti‐CD62L‐PE‐Cy7.

To assess the surface expression of α7nAChRs, freshly isolated Tcm and Tem cells were co‐stained with Alexa Fluor 647‐conjugated α‐bungarotoxin (α‐BTX; Thermo Fisher Scientific), a high‐affinity and widely used probe for α7nAChRs [38]. Cells were stained on ice with fluorochrome‐conjugated antibodies to define memory Th‐cell subsets. After washing, cells were incubated in PBS with Alexa Fluor 647–conjugated α‐BTX for 30 min at room temperature in the dark. Gating was applied to CD3^+^ CD4^+^ CD45RO^+^ CCR7^+^ cells for Tcm and CD3^+^ CD4^+^ CD45RO^+^ CCR7^−^ cells for Tem. As gating controls, cells were incubated either with PBS alone (unstained) or with unlabeled α‐BTX under identical conditions. Following incubation, cells were washed with staining buffer, and α‐BTX binding was quantified by flow cytometry. Data acquisition was performed on a FACS Verse flow cytometer, and data were analyzed using FlowJo software (BD Biosciences).

### 2.5 Quantitative Real‐Time PCR

RNA extraction, cDNA synthesis, and RT‐qPCR were performed as previously described [5]. TaqMan assays were used for human *TBX21* (Hs00894392_m1), *RORC* (Hs01076112_m1), *GATA3* (Hs00231122_m1), *BCL6* (Hs00153368_m1), and *CHRNA7* (Hs01063372_m1), with *PPIA* (Hs99999904_m1) as the housekeeping control. Amplification was carried out on a CFX96 Real‐Time System C1000 Thermal Cycler (Bio‐Rad Laboratories) with the following cycling conditions: 50°C for 2 min, 95°C for 20 s, followed by 50 cycles of 95°C for 3 s and 60°C for 30 s. Gene expression levels were normalized to the reference gene *PPIA*, and the relative expression levels of *TBX21*, *RORC*, *GATA3*, and *BCL6* were calculated using the 2^−ΔΔC^ *^t^* method. *CHRNA7* expression was also assessed using the 2^−ΔΔC^ *^t^* method, specifically for evaluating knockout efficiency. For comparisons of *CHRNA7* expression between freshly isolated Tcm and Tem subsets, relative transcript abundance was calculated as 1,000,000 × 2^−^ΔC*^t^*, where ΔC*t* = C*t* (*CHRNA7*)—Ct (*PPIA*); samples with undetermined C*t* values after 50 cycles were considered below the detection limit.

### 2.6 Western Blotting and Signal Intensity Quantification of Bands

Total memory Th cells were stimulated with 1 ng/mL PMA and 1 ng/mL ionomycin for 24 h, followed by washing and a 24 h rest in the incubator. A total of 1.5 × 10^6^ cells/mL were incubated with or without 3.125 µL/mL ImmunoCult, 10 µM nicotine, and 10 µM GTS‐21, and the cells were lysed after 15 and 60 min. Whole‐cell protein lysates were prepared as previously described [5], including lysis buffer composition, centrifugation to remove cell debris, addition of Laemmli buffer, and sample denaturation. A total of 20 µg of protein, quantified using the Detergent Compatible Protein Assay Kit II (Bio‐Rad Laboratories) and a microplate reader (BioTek, BD Bioscience), was size‐fractionated on a 10% SDS‐PAGE gel and transferred to a nitrocellulose membrane (Bio‐Rad Laboratories). The blots were blocked overnight with 5% nonfat milk (Anatol Spices, Montreal, Canada) in Tris‐buffered saline containing 0.1% Tween‐20 (TBST). The blots were incubated overnight at 4°C with primary antibodies, including monoclonal pNF‐κB p65 (Ser 529)‐specific antibody (1:500 dilution, clone 817403, Biolegend), monoclonal NF‐κB p65‐specific antibody (1:2500 dilution, clone 465003, Biolegend), and monoclonal GAPDH antibody (1:5000 dilution, clone 10B4E3, Cusabio) in TBST supplemented with 5% nonfat milk. A secondary goat anti‐mouse IgG HRP (1:3000 dilution) in 5% milk/TBST was applied for 2 h at room temperature. Chemiluminescence detection was performed using Clarity Western Enhanced Chemiluminescent substrate and analyzed with the ChemiDoc XRS+ system and Image Lab 5.1 software (Bio‐Rad). Uncropped immunoblots corresponding to these analyses are provided in Figure S2.

### 2.7 CRISPR‐Cas9 Ribonucleoprotein Knockout of CHRNA7 in Memory Th Cells

CRISPR‐Cas9 employing single guide RNAs (sgRNAs) was used to knockout *CHRNA7* in total human memory Th cells following an adapted protocol [5]. Briefly, gene editing was carried out using Cas9 2NLS nuclease and sgRNAs targeting *CHRNA7*, with *TRAC*‐targeting sgRNAs serving as a positive control and a nontargeting sgRNA as a negative control (Synthego, Menlo Park, CA). The payload sequences for the multi‐sgRNAs are attached in Table S1. Total memory Th cells were mixed with either scrambled sgRNA or multi‐sgRNAs targeting *CHRNA7* or *TRAC*, and electroporated using the Neon transfection system (ThermoFisher Scientific). Knockout efficiency was assessed after 4 days via fluorescence‐based cell counting for TRAC, calculated by dividing the number of living cells with low fluorescence by the total number of living cells and multiplying by 100, and RT‐qPCR analysis for *CHRNA7*. Surface expression of α7nAChRs was also evaluated as part of knockout validation by staining cells with Alexa Fluor 647‐conjugated α‐BTX. For functional assays, memory Th cells (0.3 × 10^6^ per well) were plated in 96‐well U‐bottom plates and left unstimulated or stimulated for five days with nicotine or GTS‐21, followed by collection of cell‐free supernatants for ELISA analysis.

### 2.8 Statistical Analysis

The results are expressed as mean values ± SD. For paired comparisons between Tcm and Tem, a two‐tailed paired Student's *t*‐test was used. For group comparisons, one‐way or two‐way ANOVA followed by Tukey's multiple comparisons test was applied. Statistical significance was defined as *p < 0.05, **p < 0.01, ***p < 0.001, ****p < 0.0001. All analyses were performed using GraphPad Prism software, version 6.0.

## 3 Results

### 3.1 Nicotine and GTS‐21 Reduced IFN‐γ, IL‐17A, and IL‐4, but Not Proliferation or Viability of Total Human Memory Th Cells

To assess the regulatory effects of nicotine and GTS‐21 on cytokine secretion in total human memory Th cells, IFN‐γ, IL‐17A, and IL‐4 levels were measured in both culture supernatants and intracellularly after five days of incubation. Nicotine and GTS‐21 each reduced IFN‐γ, IL‐17A, and IL‐4 secretion compared with the activated control group (Figure 1A–C). To determine whether the observed effects of nicotine and GTS‐21 on total memory Th cells were mediated through α7nAChR, CRISPR/Cas9 gene editing was used to induce a loss‐of‐function mutation in *CHRNA7*. The efficiency of *CHRNA7* knockout was confirmed by RT‐qPCR, demonstrating an absence of *CHRNA7* mRNA in the edited group (Figure S5A). Consistent with reduced gene expression, surface α7nAChR levels were markedly diminished as measured by α‐BTX binding (Figure S5B). *TRAC* editing served as a positive control, yielding 89.5% knockout efficiency by flow cytometry (Figure S5C,D). In *CHRNA7* knockout memory Th cells, nicotine failed to suppress IFN‐γ, IL‐17A, and IL‐4 secretion, with cytokine levels remaining similar to those in the activated control group. Application of GTS‐21 retained partial inhibitory activity even in the *CHRNA7* knockout cells (Figure 1D–F). These findings show that nicotine failed to suppress cytokine secretion following *CHRNA7* disruption, whereas GTS‐21 retained a partial inhibitory effect. To determine if the proportions of Th subsets were altered, intracellular cytokine expression was determined by flow cytometry. The data showed that both nicotine and GTS‐21 reduced the proportion of memory Th cells expressing IFN‐γ and IL‐17A, whereas the frequency of IL‐4‐expressing cells was unchanged by nicotine and increased following GTS‐21 treatment (Figure 2A–O). The overall reduction in cytokines suggested that either cellular proliferation was reduced or cell death was increased. However, nicotine and GTS‐21 treatment did not change the proliferation index or division index compared with activated controls (Figure 2P,R). Moreover, nicotine and GTS‐21 did not affect the viability of memory Th cells (Figure 2S,T). Together, these results indicate that the effects of nicotine and GTS‐21 occur independently of cellular proliferation or cytotoxicity.

### 3.2 Nicotine and GTS‐21 Downregulated *TBX21*, *RORC*, and *GATA3* mRNA Levels and Phosphorylation of NF‐κB p65 in Total Memory Th Cells

To evaluate the transcriptional effects of nicotine and GTS‐21 on total memory Th cells, the mRNA expressions of *TBX21* (Th1), *RORC* (Th17), and *GATA3* (Th2) were evaluated. Nonactivated cells expressed the Th transcription factors, which was expected since these are purified memory Th cells. Nicotine reduced *TBX21*, *RORC*, and *GATA3* mRNA expression, with decreases of 0.35‐fold, 0.40‐fold, and 0.08‐fold, respectively, compared with the activated control condition. Similarly, GTS‐21 treatment resulted in 0.58‐fold, 0.47‐fold, and 0.34‐fold reductions in *TBX21*, *GATA3*, and *RORC* expression, respectively (Figure 3A–C). These data indicate that both nicotine and GTS‐21 suppress the expression of transcription factors involved in Th1, Th17, and Th2 phenotypes, potentially impacting the development and function of these Th cell subsets. To further examine downstream pathways, the effects of nicotine and GTS‐21 on NF‐κB p65 phosphorylation were examined. Western blot analysis demonstrated a time‐dependent modulation of NF‐κB p65 phosphorylation following T cell activation. Memory Th cell activation led to an increase in NF‐κB p65 phosphorylation, which remained unchanged in the presence of nicotine or GTS‐21 at 15 min. At 60 min postactivation, both nicotine and GTS‐21 significantly suppressed NF‐κB p65 phosphorylation compared with the activated condition at the same time point (Figure 3D,E). These findings show that the transcriptional inhibitory effects of nicotine or GTS‐21 on NF‐κB p65 phosphorylation occur as early as 60 min postactivation.

### 3.3 Nicotine and GTS‐21 Reduced Th1, Th17, and Th2 Subset Frequencies in Central Memory Th Cells, But Not in Effector Memory Th Cells

Having established the effects of nicotine and GTS‐21 on total memory Th cells, we next asked whether specific memory subsets were differentially affected using CCR7 expression to define Tcm versus Tem. To this end, chemokine receptor expression was assessed to distinguish Th1 (CXCR3^+^ CCR6^−^), Th17 (CXCR3^−^ CCR6^+^), and Th2 (CXCR3^−^ CCR6^−^ CCR4^+^) subsets within Tcm (CD4^+^ CD45RA^−^ CCR7^+^) and Tem (CD4^+^ CD45RA^−^ CCR7^−^). Memory Th cells were gated as CD45RA^−^, corresponding to CD45RO^+^ memory cells as previously validated [5]. The flow cytometry gating strategy is shown in Figure 4A–E, illustrating the classification of Th subsets based on chemokine receptor expression. Both nicotine and GTS‐21 treatment reduced the percentages of Th1, Th17, and Th2 subsets within the Tcm population (Figure 4F–H). However, no changes were observed in the distribution of Th1, Th2, and Th17 subsets among Tem (Figure 4I–K). These findings indicate that nicotine and GTS‐21 preferentially altered chemokine receptor‐defined Th subset distributions within the Tcm population.

### 3.4 Separated Central Memory Th Cells Express Higher Levels of α7nAChRs Than Effector Memory Th Cells; Nicotine Suppressed Cytokines in Central Memory Th Cells, While GTS‐21 Extended Suppression to Effector Memory Th Cells

In the previous section, memory Th subsets were distinguished within total memory Th cells by CCR7‐based flow cytometric gating. To investigate α7nAChR availability and expression, we next physically separated Tcm and Tem subsets using a subset‐specific isolation kit based on CD62L. The purity of Tcm (CD3^+^ CD4^+^ CD45RO^+^ CD62L^+^) and Tem (CD3^+^ CD4^+^ CD45RO^+^ CD62L^−^) fractions was confirmed to be over 94% by flow cytometry (Figure 5A–D). We assessed both surface binding of Alexa Fluor 647‐labeled α‐BTX and *CHRNA7* mRNA expression in freshly isolated Tcm and Tem subsets. The gating strategy for α‐BTX^+^ cells is shown in Figure 5E–G. The frequency of α‐BTX^+^ cells was significantly higher in Tcm than in Tem (Figure 5H). This is consistent with a greater surface receptor availability of α7nAChR. *CHRNA7* transcript abundance was detectable but remained near the lower limit of assay sensitivity. When normalized to *PPIA*, *CHRNA7* expression was markedly greater in Tcm compared with Tem (Figure 5I). In Tem, *CHRNA7* amplification did not reach the detection threshold within 50 cycles in any donor and was therefore considered nondetectable. Together, these findings indicate that α7nAChR is more prominently expressed in Tcm than Tem at both the protein and transcript levels.

Next, cytokine responses to nicotine and GTS‐21 were examined in separate subsets. In Tcm, activation induced robust secretion of IFN‐γ, IL‐17A, and IL‐4, all of which were suppressed by nicotine; GTS‐21 produced a similar inhibitory effect (Figure 5J–L). In contrast, cytokine secretion from Tem was not significantly altered by nicotine, whereas GTS‐21 suppressed IFN‐γ, IL‐17A, and IL‐4 production from Tem (Figure 5M–O). These findings show that nicotine suppresses cytokine secretion predominantly in Tcm, whereas GTS‐21 suppresses cytokine secretion in both Tcm and Tem subsets.

### 3.5 Nicotine Lowered IL‐21, *BCL6*, and Inhibited CD40L in Separated Central Memory Th Cells but Not in Effector Th Cells; GTS‐21 Extended Its Effect to Effector Th Cells

We examined the impact of nicotine and GTS‐21 on the Tfh‐associated cytokine IL‐21, the transcription factor *BCL6*, and the early activation marker CD40L in separated Tcm and Tem subsets.

In Tcm, both nicotine and GTS‐21 reduced IL‐21 and *BCL6* relative to activated controls (Figure 6A,B). Representative purity gates for Tcm are shown in Figure 6C, and Tfh (CXCR5^+^) and non‐Tfh (CXCR5^−^) populations were defined as illustrated in Figure 6D. Within Tcm, T cell activation increased CD40L expression in both Tfh and non‐Tfh cells, whereas treatment with either agonist reduced CD40L compared with activated controls (Figure 6G,H).

In contrast, Tem displayed a differential pattern of responsiveness. Nicotine had no effect on IL‐21 or *BCL6*, whereas GTS‐21 suppressed both readouts (Figure 6I,J). Representative purity gates for Tem are shown in Figure 6K, and Tfh and non‐Tfh populations were defined as shown in Figure 6L. Consistent with the cytokine data, nicotine did not alter CD40L expression in Tfh or non‐Tfh Tem cells, whereas GTS‐21 reduced CD40L in both Tfh and non‐Tfh subsets (Figure 6O,P).

Taken together, these findings indicate that nicotine and GTS‐21 attenuate Tfh‐associated readouts, including IL‐21, *BCL6*, and CD40L, within the Tcm compartment. In contrast, GTS‐21, but not nicotine, reduced these readouts in Tem.

## 4 Discussion

Memory Th cells sustain protective immunity but can also drive chronic inflammation, highlighting the need for mechanisms that precisely regulate their effector programs. It was previously unclear how nicotinic receptor‐associated signaling regulates Th1, Th17, Th2, and Tfh programs, as well as CD40L expression, within memory Th cells, particularly across Tcm and Tem subsets. We addressed this gap by defining how nicotine and GTS‐21 reshape cytokine output, transcription‐factor programs, chemokine‐receptor profiles, and CD40L in human memory Th cells.

We found that in total human memory Th cells, nicotine and GTS‐21 each reduced IFN‐γ, IL‐17A, and IL‐4. Consistent with our findings, prior studies reported reduced IFN‐γ levels in PBMCs following nicotine exposure from cigarette smoke extracts [39] and inhibition of OVA‐induced IFN‐γ, IL‐4, and IL‐17 production by GTS‐21 in spleen cells from DO11.10 TCR transgenic mice [40]. In contrast, nicotine treatment enhanced IL‐4 and *GATA3* expression in Th2‐differentiated cells from rheumatoid arthritis patients [41, 42] and in experimental autoimmune encephalomyelitis models [4]. These discrepancies likely reflect differences in cellular context and immune environment. Prior studies were conducted in chronic inflammatory disease settings characterized by persistent antigen exposure and elevated proinflammatory cytokines, whereas our study examined healthy human memory Th cells. In this context, signaling engaged by agonists appears to promote a distinct mode of regulation, characterized by coordinated suppression of Th1‐, Th2‐, and Th17‐associated cytokines, consistent with pan‐suppression rather than lineage deviation toward Th2 responses. In line with this interpretation, we observed reduced NF‐κB p65 phosphorylation together with decreased expression of lineage‐defining transcription factors, indicating attenuation of early activation‐associated signaling. Memory Th cells are poised for rapid effector responses and may therefore be particularly sensitive to modulation of proximal signaling pathways, resulting in coordinated suppression of cytokine output across Th subsets.

Our data indicate that intracellular IL‐4 positivity was unchanged (nicotine) or increased (GTS‐21) despite reduced IL‐4 secretion. Because intracellular cytokine staining was performed after brief PMA/ionomycin restimulation in the presence of brefeldin A, it reflects the frequency of cells capable of producing IL‐4 under maximal stimulation rather than cumulative cytokine output over the 5‐day culture. Thus, reduced IL‐4 secretion can coexist with preserved IL‐4 positivity if treatment lowers per‐cell IL‐4 output or dampens overall activation. The accompanying reduction in *GATA3* mRNA is consistent with dampening of the Th2 transcriptional program, without necessarily eliminating inducible IL‐4 production.

To determine whether the effects of nicotine and GTS‐21 are dependent on α7nAChR, we created *CHRNA7*‐deficient memory Th cells. In these cells, nicotine failed to suppress cytokine production, supporting the conclusion that its immunosuppressive effects are α7nAChR‐dependent. This is consistent with findings from *CHRNA7*‐deficient mice, where splenocytes secreted higher IFN‐γ levels compared with wild‐type controls [34], and nicotine failed to modulate TNF, IFN‐γ, IL‐17, and IL‐4 production in T cells from *CHRNA7*‐deficient experimental autoimmune encephalomyelitis models [4]. Although GTS‐21 has been described as a partial agonist of α7nAChR [31, 32], it retained inhibitory activity following *CHRNA7* disruption, indicating that its effects are not fully explained by α7nAChR signaling alone. Consistent with α7nAChR‐independent actions, GTS‐21 inhibited TNF and IL‐6 in α7^−^/^−^ macrophages [43], and its cytokine‐suppressive effects in human leukocytes were not reversed by α7nAChR antagonists or nonselective nAChR blockers [44]. One potential α7nAChR‐independent mechanism that could contribute to the residual inhibitory effect of GTS‐21 is antagonism of 5‐hydroxytryptamine type 3 (5‐HT_3_) receptors. T cells express functional 5‐HT_3_ receptors, and serotonin signaling through this pathway promotes T‐cell activation, whereas 5‐HT_3_ blockade dampens T‐cell responses [45, 46]. In vitro studies have shown that GTS‐21 can act as a 5‐HT3 receptor antagonist at higher concentrations [47, 48]. These data indicate that GTS‐21 can engage α7nAChR‐independent mechanisms.

Nicotine and GTS‐21 inhibited transcription factors *TBX21*, *RORC*, *GATA3*, and NF‐κB p65 phosphorylation in human memory Th cells. This observation aligns with previous studies where nicotine inhibited NF‐κB activation in human monocytes [49] and GTS‐21 inhibited NF‐κB transcriptional activity in murine microglia cells [36]. These findings collectively suggest that nicotine or GTS‐21 can modulate NF‐κB signaling across different immune cell types and species. Given the central role of NF‐κB p65 in driving *TBX21* and *RORC* expression, its inhibition is associated with the observed reduction in IFN‐γ and IL‐17A production by Th1 and Th17 cells. In the context of Th2 regulation, the expression of IL‐4 and its upstream transcription factor *GATA3* has been shown to depend on NF‐κB p50, functioning as part of a p50/p65 heterodimer [13, 50]. Therefore, inhibition of NF‐κB p65 phosphorylation by nicotine and GTS‐21 could limit the formation of active p50/p65 heterodimers, thereby attenuating *GATA3*‐driven transcription and downstream IL‐4 expression. These findings support a model in which inhibition of NF‐κB p65 by nicotine and GTS‐21 contributes to the coordinated downregulation of *TBX21*, *GATA3*, and *RORC*, resulting in suppressed cytokine production by Th1, Th2, and Th17 cells.

We conducted further analyses to determine whether Tcm and Tem subsets differ in their susceptibility to modulation by nicotinic agonists. Tcm subsets traffic to the secondary lymphoid tissues predominantly and possess high proliferative potential. In contrast, Tem cells circulate through peripheral tissues and are terminally differentiated [51]. We found that nicotine and GTS‐21 preferentially reduced the proportions of Th1 (CXCR3^+^ CCR6^−^ CCR4^−^), Th2 (CXCR3^−^ CCR6^−^ CCR4^+^), and Th17 (CXCR3^−^ CCR6^+^ CCR4^+^) subsets within Tcm (CCR7^+^) cells, while no significant changes were observed in Tem (CCR7^−^) cells.

To address whether differential responsiveness to nicotine is linked to α7nAChR availability, we evaluated α‐BTX binding and *CHRNA7* expression in freshly isolated Tcm and Tem cells. Both *CHRNA7* transcript levels and surface α‐BTX binding were markedly higher in Tcm compared with Tem, supporting the hypothesis that reduced receptor expression underlies the diminished responsiveness of Tem to nicotine. Previous studies reported that *CHRNA7* expression is undetectable in freshly isolated CD4^+^ T cells but is induced upon TCR‐mediated activation [3]. Our findings extend this observation by demonstrating that Tcm cells maintain detectable *CHRNA7* expression even in the absence of stimulation, albeit at low levels as indicated by relatively high C*t* values. This low basal expression likely primes Tcm cells for α7nAChR‐mediated regulation, providing a plausible explanation for their selective responsiveness to nicotine. In contrast, the lack of *CHRNA7* detection in Tem suggests insufficient receptor availability to mediate this effect. While the sensitivity of qPCR may limit the detection of very low transcript levels, surface staining with α‐BTX further confirmed subset‐specific differences in receptor availability at the protein level. It is possible that Tem have *CHRNA7* transcripts below the detection threshold, but still sufficient to yield expression of the receptor on the cell surface. Together, these results support the idea that intrinsic differences in α7nAChR expression contribute to the differential response of memory Th subsets to nicotinic modulation. An additional explanation for this selective effect is the greater functional plasticity of Tcm, potentially making them more responsive to α7nAChR‐mediated signaling in secondary lymphoid tissues, whereas Tem are less responsive due to their more differentiated phenotype [52].

Building on these subset‐specific differences in α7nAChR availability, we next examined how nicotinic signaling functionally shapes cytokine and helper programs across memory Th subsets within the Tcm and Tem compartments. We found that Tcm were more sensitive than Tem to modulation by nicotine and GTS‐21. Nicotine suppressed IFN‐γ, IL‐17A, and IL‐4 in Tcm but not Tem, whereas GTS‐21 suppressed these cytokines in both Tcm and Tem. The more extensive suppressive effects observed with GTS‐21 are consistent with prior human leukocyte assays showing stronger inhibition of inflammatory cytokines by GTS‐21 than nicotine in PBMCs [44]. Moreover, IL‐21, *BCL6*, and CD40L were reduced in Tcm by nicotine, but GTS‐21 extended this suppression to both Tcm and Tem. Because IL‐21, *BCL6*, and CD40L are canonical determinants of Tfh help and germinal‐center function, reductions in these readouts would be expected to diminish B cell help and class‐switching capacity [53, 54]. CD40L was reduced in CXCR5^−^ non‐Tfh memory Th cells after treatment with nicotine in Tem, and after treatment with GTS‐21 in both Tcm and Tem. The most efficient B cell helpers are the Tfh lineage cells that co‐express CXCR5 and produce high IL‐21, localizing to follicles; however, non‐Tfh memory T cells can promote antibody responses in extrafollicular settings or early phases of immune responses [23]. These data indicate a cholinergic brake on extrafollicular T‐B help outside of germinal centers. This heightened sensitivity of Tcm to nicotine is consistent with their higher α7nAChR availability relative to Tem.

This study shows for the first time that nicotine preferentially inhibited human memory Th1, Th2, Th17, and Tfh‐associated responses in Tcm but not Tem, accompanied by reduced cytokine production, lower expression of lineage‐defining transcription factors, shifts in chemokine receptor‐defined subset distributions, and reduced CD40L. This subset‐selective sensitivity is consistent with higher α7nAChR availability in Tcm compared with Tem. GTS‐21 produced similar suppressive effects and extended these effects to Tem. Together, these findings identify an α7nAChR‐dependent checkpoint engaged by nicotine that preferentially regulates Tcm function and may represent a therapeutic entry point to limit pathogenic recall responses in chronic immune‐mediated disease, particularly in settings where Tcm contribute to disease recurrence.

### 4.1 Data Limitations and Perspectives

A limitation of this study is that alternative receptor pathways that may contribute to the α7nAChR‐independent effects of GTS‐21 were not directly examined. Future studies incorporating selective 5‐HT_3_ receptor agonists or antagonists, as well as complementary pharmacological approaches, will be informative in defining the mechanisms underlying GTS‐21–mediated immunomodulation.

## Author Contributions

Conceptualization: F.G.; Research design: F.G., P.J.D.; Methodology: F.G., M.H.; Software: F.G.; Resources: P.J.D.; Investigation: F.G., M.H., N.R., J.S.C., S.H., A.C., M.Kaz., M.Kar., A.P.; Data analysis: F.G.; Validation: F.G.; Data curation: F.G., P.J.D.; Visualization: F.G.; Writing, original draft preparation: F.G.; Writing, review and editing: P.J.D., S.C.C.S.; Funding acquisition: P.J.D. All authors have read and agreed to the published version of the manuscript.

## Conflicts of Interest

The authors declare no conflicts of interest.

## Supporting information




**Supporting File**: eji70177‐sup‐0001‐SuppMat.pdf.

## Data Availability

The data that support the findings of this study are available from the corresponding author upon reasonable request.
