# Supplementary material for: Nicotine Suppresses Human Memory Th Cell Subsets With Preferential Effects on Central Memory Th Cells in an α7 Nicotinic Acetylcholine Receptor‐Dependent Manner
Source: Eur J Immunol. 2026 Apr 2;56(4):e70177. doi: 10.1002/eji.70177 (PMC13047356; doi:10.1002/eji.70177)
Supplement: Supplementary file 1 — Supporting File: eji70177‐sup‐0001‐SuppMat.pdf. [file EJI-56-e70177-s001.pdf]

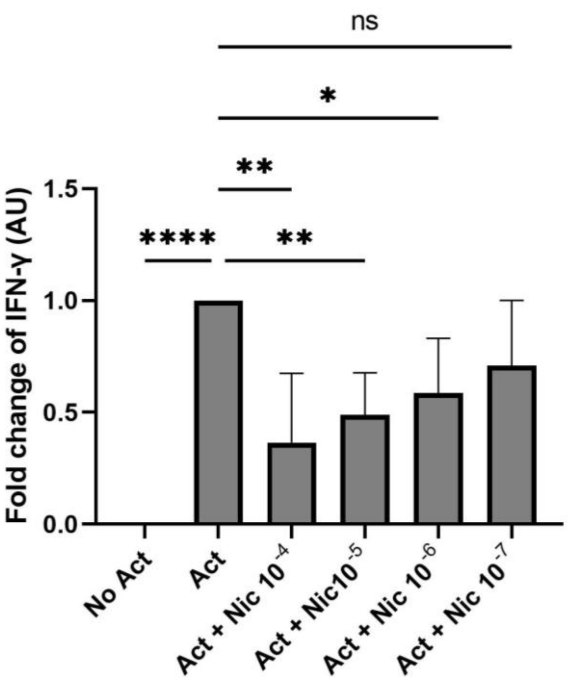

Supplemental Fig. 1. Dose-dependent effect of nicotine on IFN-γ secretion in activated PBMCs. PBMCs were unstimulated (No Act), activated (Act), or activated in the presence of decreasing concentrations of nicotine (10^{-4}-10^{-7} M). IFN-γ levels in culture supernatants were measured by ELISA after 5 days. Nicotine concentrations from 10^{-4} M to 10^{-6} M consistently suppressed IFN-γ production, supporting the use of 10 µM in subsequent experiments to ensure robust and reproducible effects. Data are presented as mean ± SD from five independent participants. Group comparisons were analyzed using one-way ANOVA with Tukey's post hoc test for multiple comparisons.

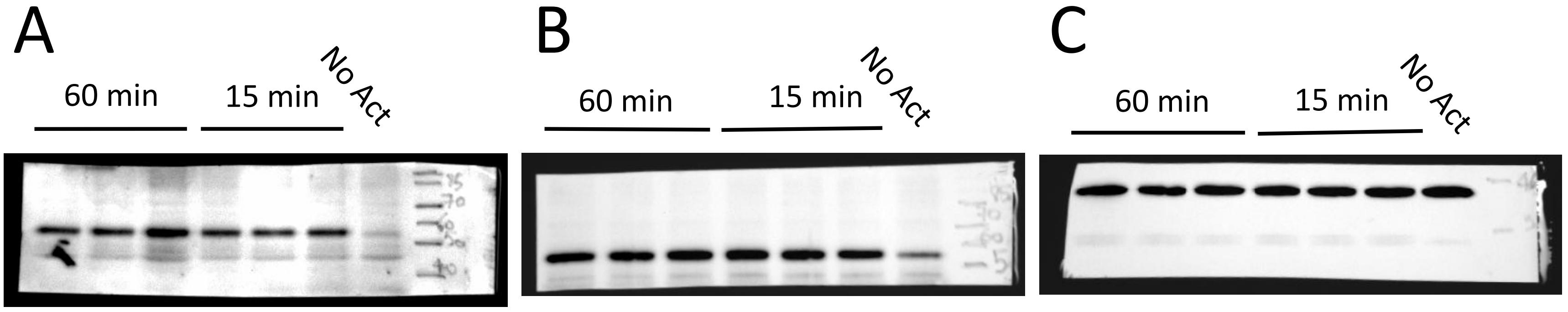

Supplemental Fig. 2. Uncropped western blot images corresponding to Fig. 3D (pNF-κB p65, total NF-κB p65, and GAPDH). (A-C) Raw immunoblots showing (A) phosphorylated NF-κB p65 (pNF-κB p65, ~60 kDa), (B) total NF-κB p65 (~65 kDa), and (C) GAPDH (~37 kDa) as a loading control. Blots in panels A-C were rotated 180° for presentation in the main figure. Molecular weight markers were manually annotated directly onto the membrane using a pencil prior to blocking, based on visible bands from a prestained protein ladder observed after Ponceau S staining. For panels A and B, the membrane was cut horizontally following protein transfer, with the upper portion used for detection of pNF-κB p65 or total NF-κB p65 and the lower portion used for GAPDH probing.

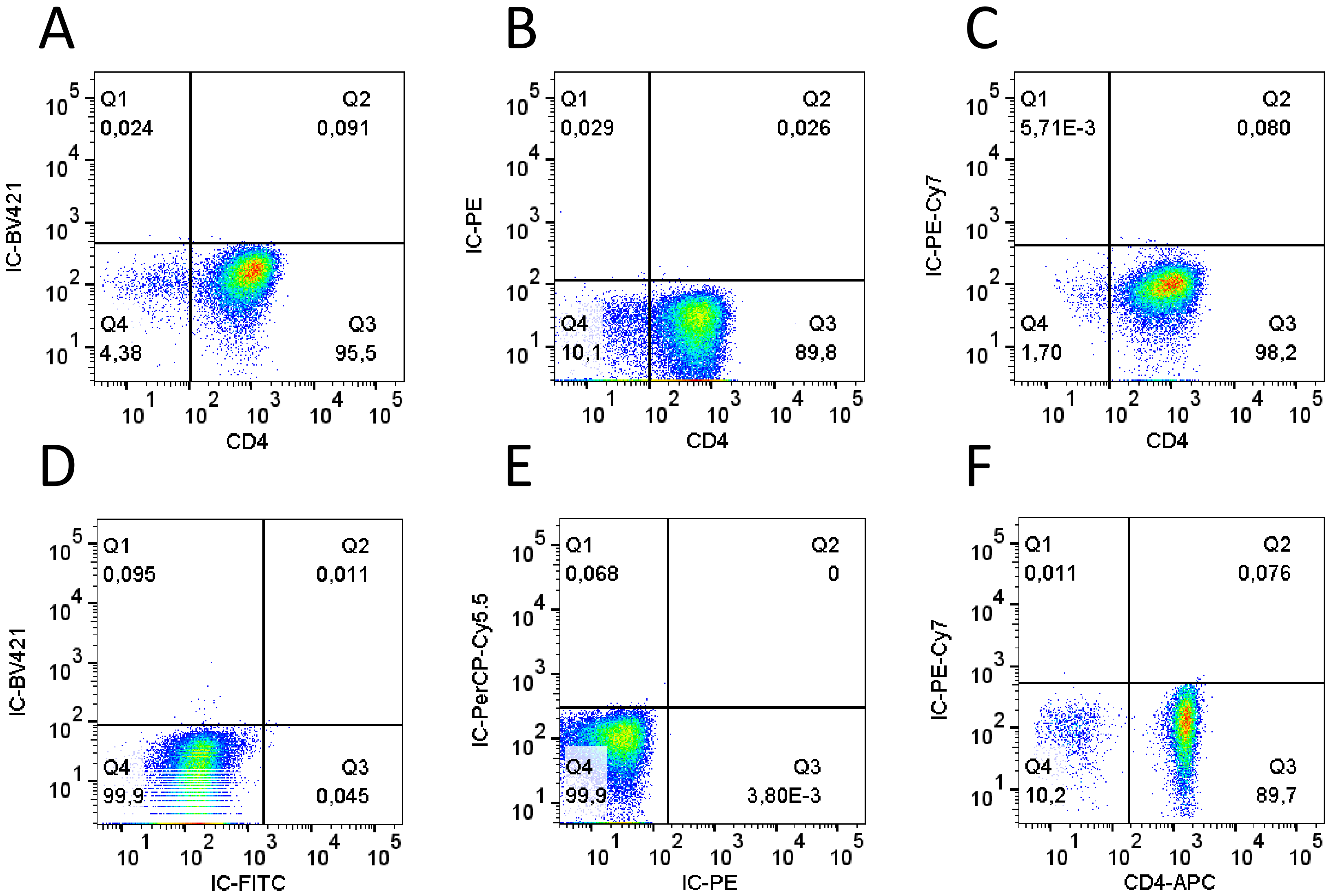

Supplemental Fig. 3. Flow cytometry plots illustrating isotype control staining for intracellular cytokines and chemokine receptors. (A-C) Isotype controls for IFN-γ (BV421), IL-17A (PE), and IL-4 (PE-Cy7). (D-F) Isotype controls for CCR7 (BV421), CD45RA (FITC), CCR6 (PE), CXCR3 (PerCP-Cy5.5), and CCR4 (PE-Cy7).

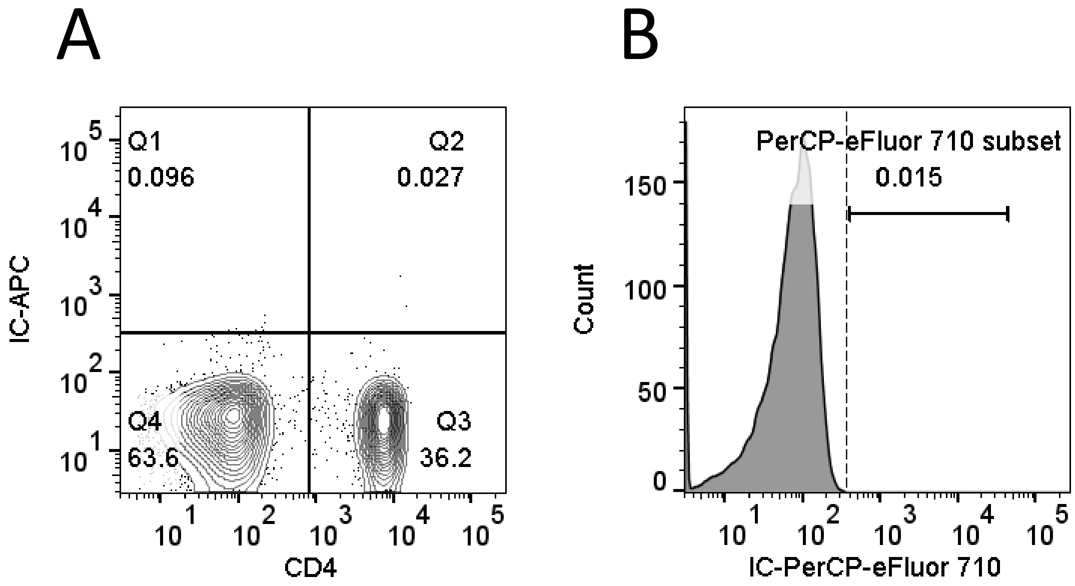

Supplemental Fig. 4. Flow cytometry plots illustrating isotype control staining for CXCR5 and CD40L in Tcm and Tem subsets. (A) Isotype controls for CXCR5 (APC) and (B) CD40L (PerCP-eFluor 710).

Supplemental Table 1: Payload sequences of multi-guide sgRNAs for targeting *TRAC* and *CHRNA7* genes

Human *TRAC* multi-guide sgRNAs	sgRNA 1: 5'-CUCUCAGCUGGUACACGGCA-3'
	sgRNA 2: 5'-GAGAAUCAAAAUCGGUGAAU-3'
	sgRNA 3: 5'-ACAAAACUGUGCUAGACAUG-3'
Human *CHRNA7* multi-guide sgRNAs	sgRNA 1: 5'- AACAUGCGCUGCUCGCCGGG-3'
	sgRNA 2: 5'- AGGCAGUGGCUUUACCGUGC-3'
	sgRNA 3: 5'- CCUCUGUGCGCCCCGCGCCU-3'

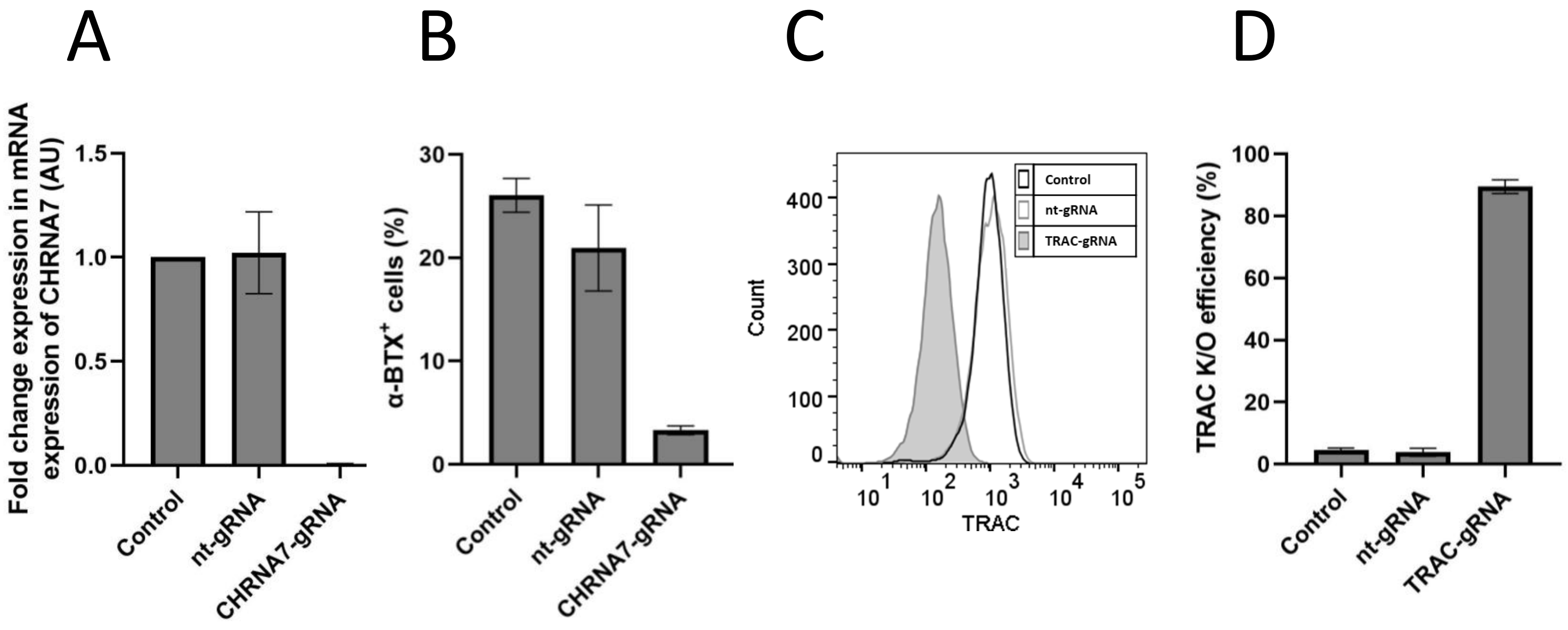

Supplemental Fig. 5. *CHRNA7* Knockout Confirmation with *TRAC* Editing Control.
(A) *CHRNA7* mRNA expression was quantified by qPCR in control cells, non-targeting guide RNA (nt-gRNA, CRISPR control), and *CHRNA7*-targeting gRNA (*CHRNA7*-gRNA) groups. (B) Surface expression of α7nAChRs was assessed by flow cytometric detection of Alexa Fluor 647–labeled α-BTX binding in control, nt-gRNA, and C*HRNA7*-gRNA groups. Gating for α-BTX$^+$ cells was performed as described in Fig. 5 E-G. (C) A representative histogram of *TRAC* expression is shown as a positive control for CRISPR editing efficiency, comparing control, nt-gRNA, and *TRAC*-targeting gRNA (*TRAC*-gRNA) groups. (D) *TRAC* knockout efficiency was calculated as the percentage of viable cells with reduced fluorescence.
